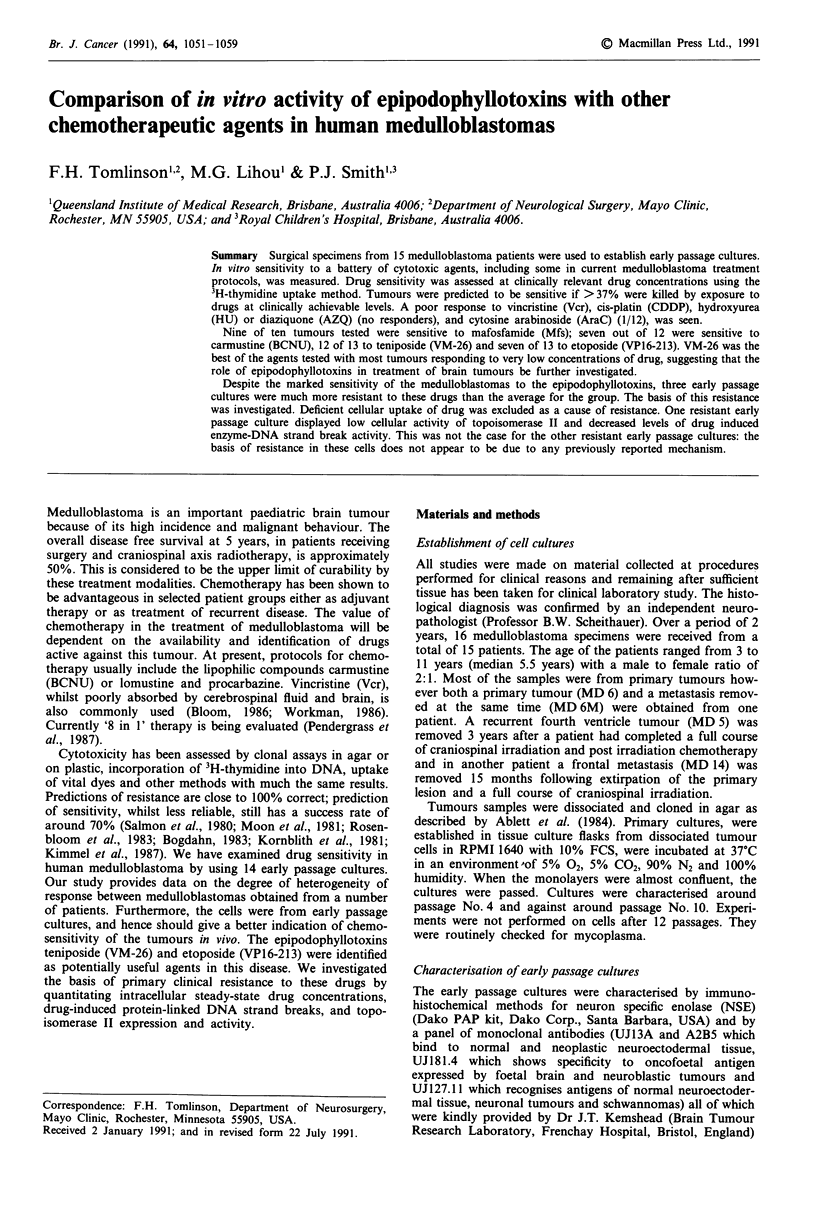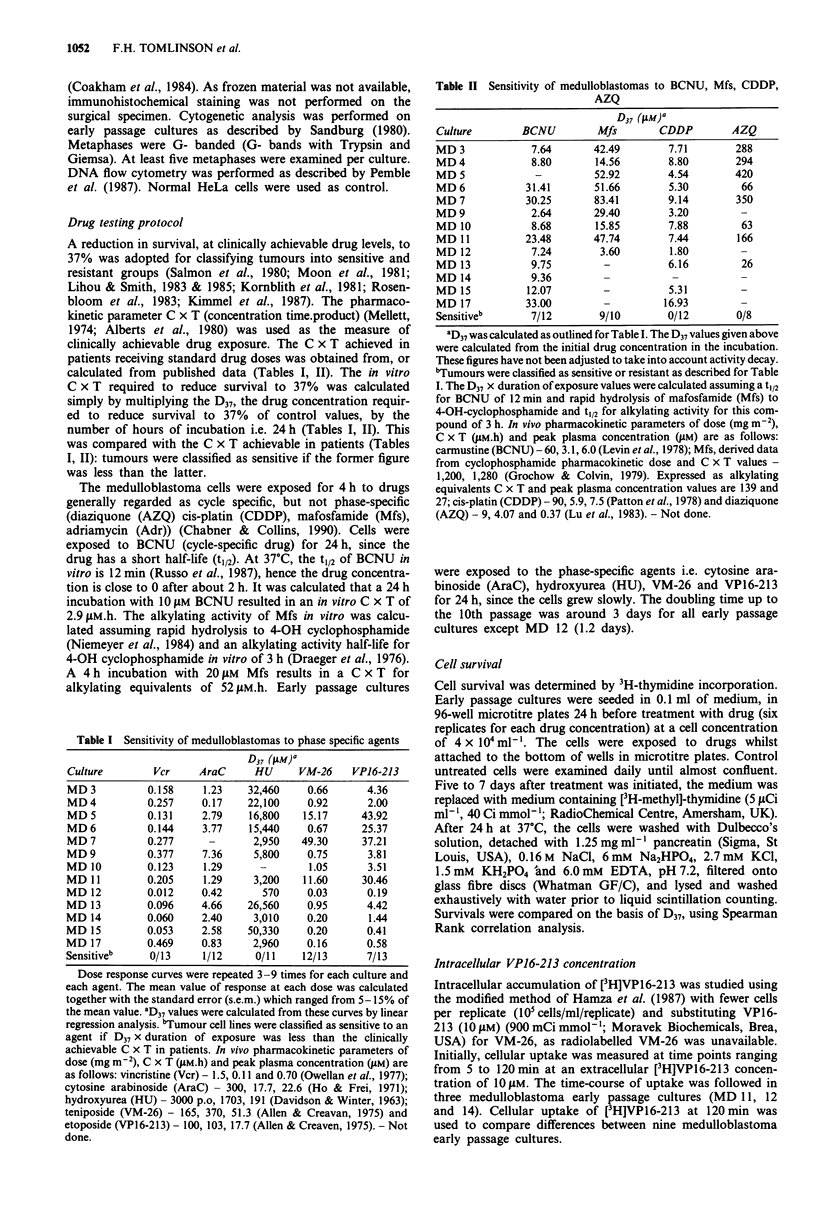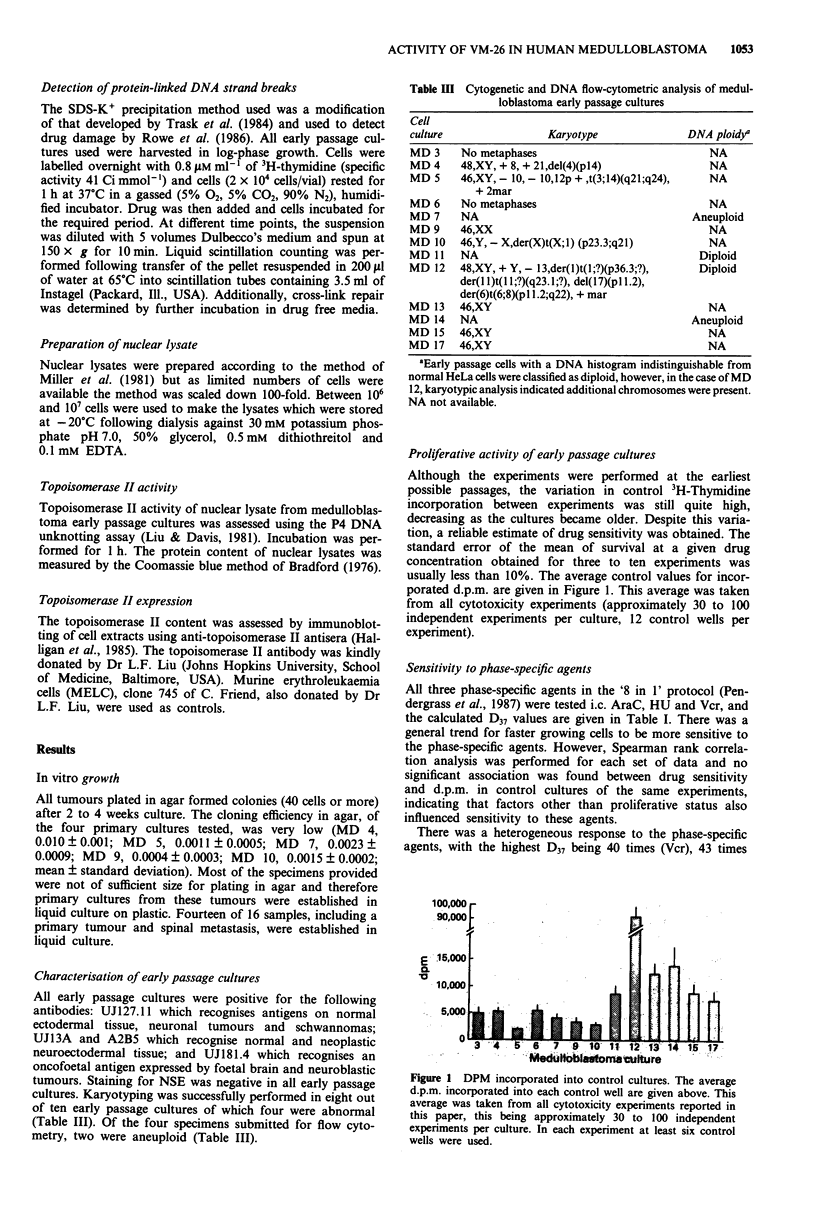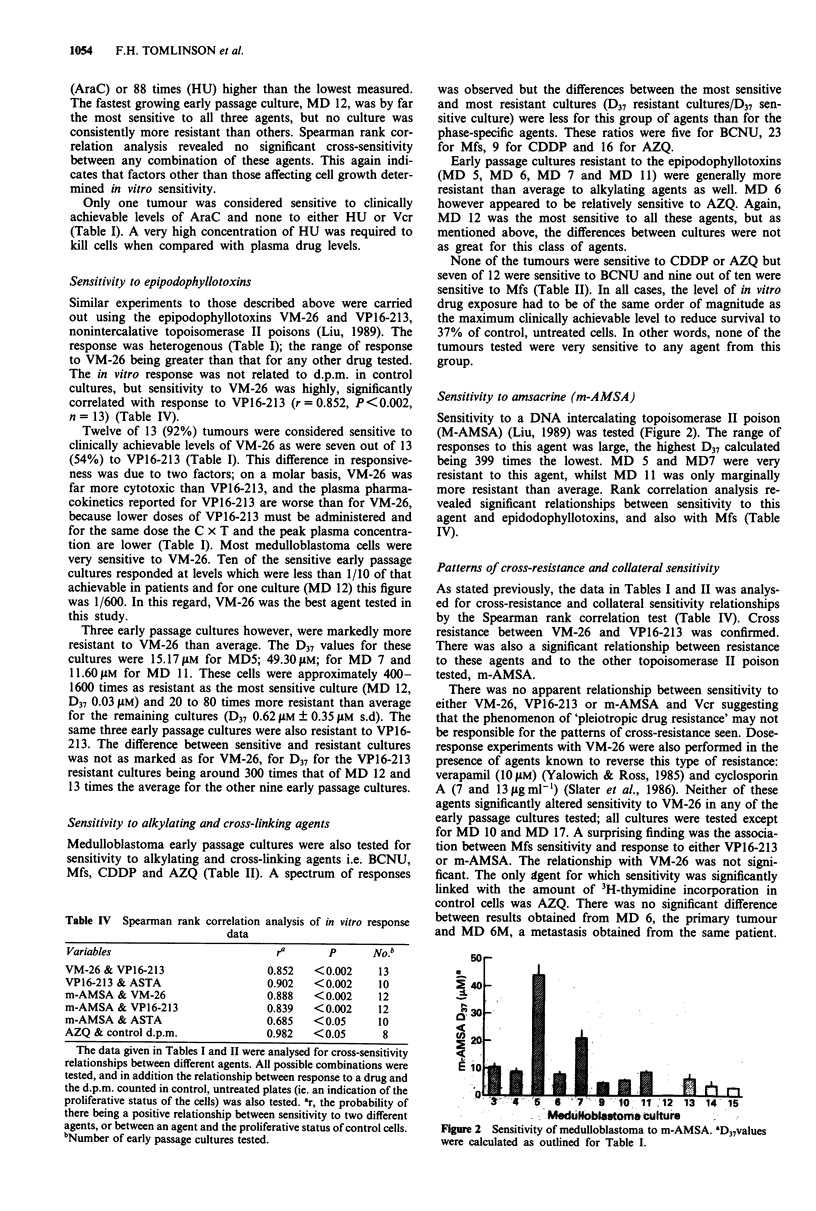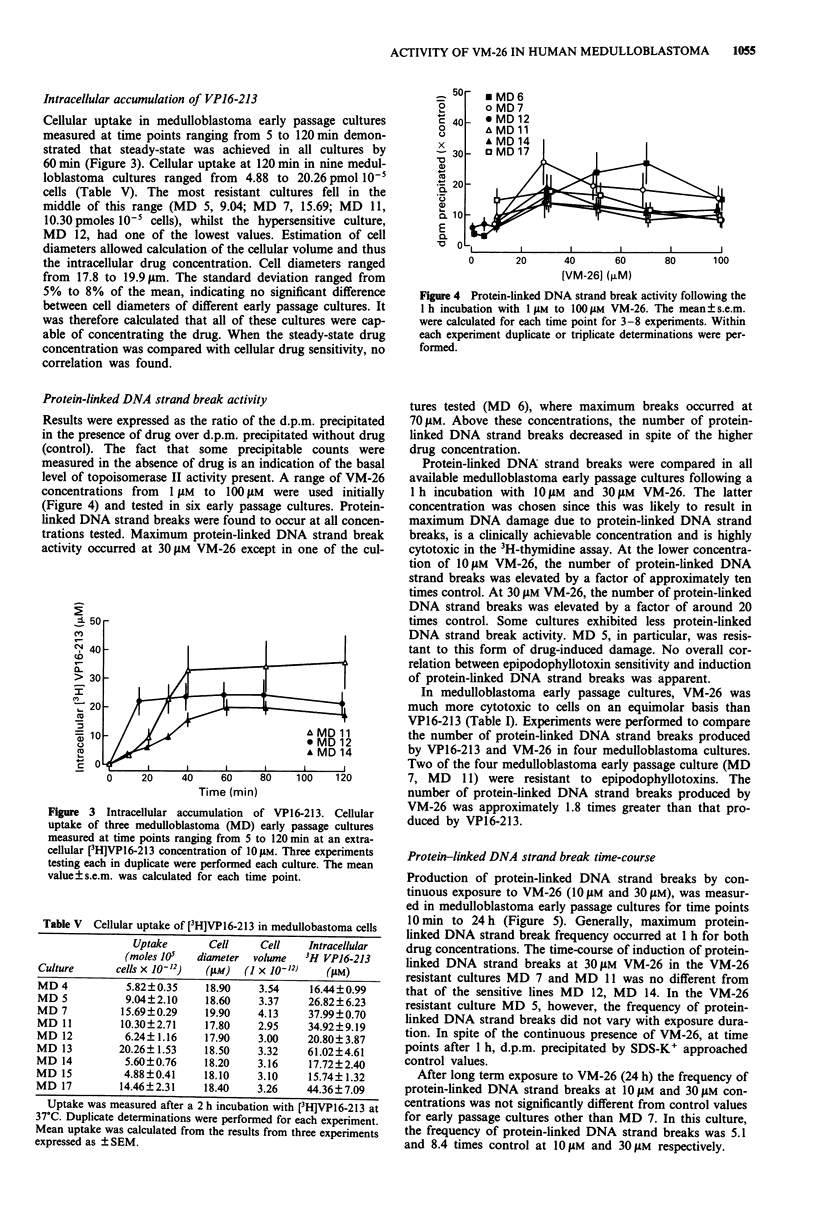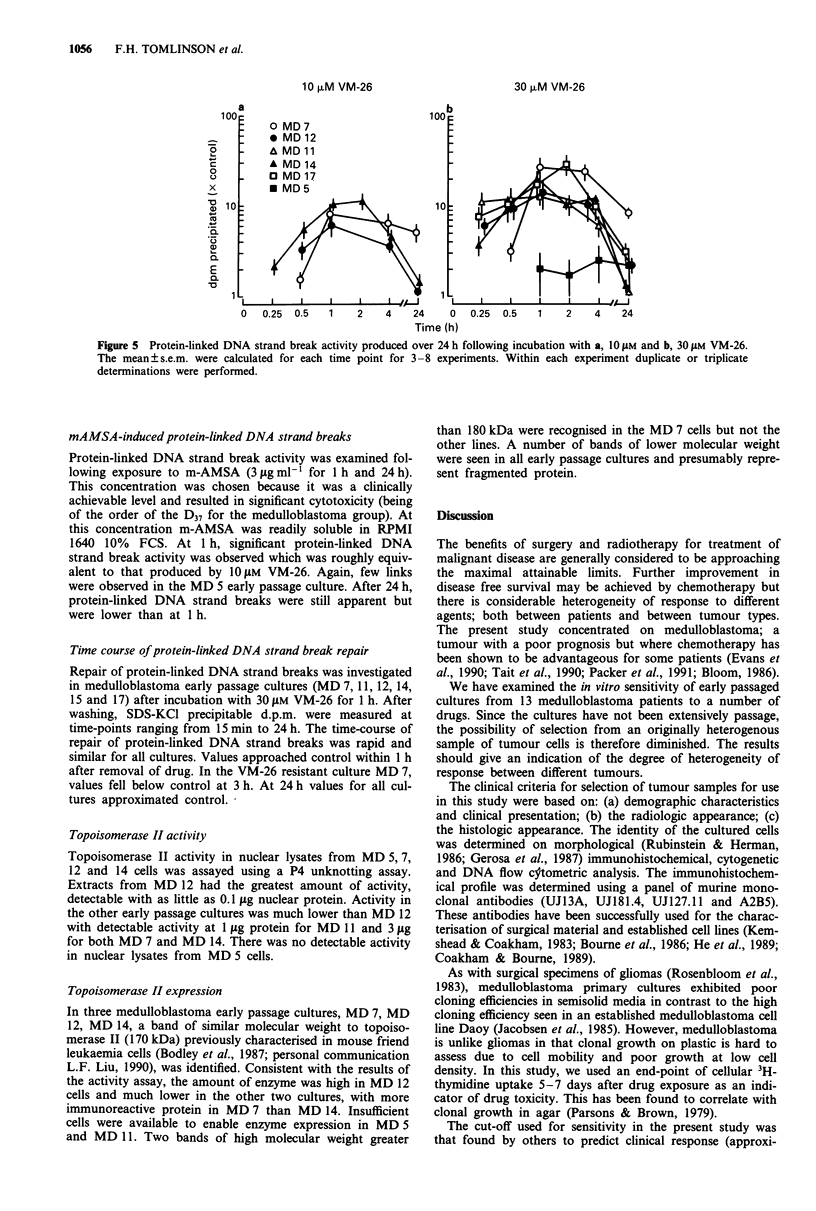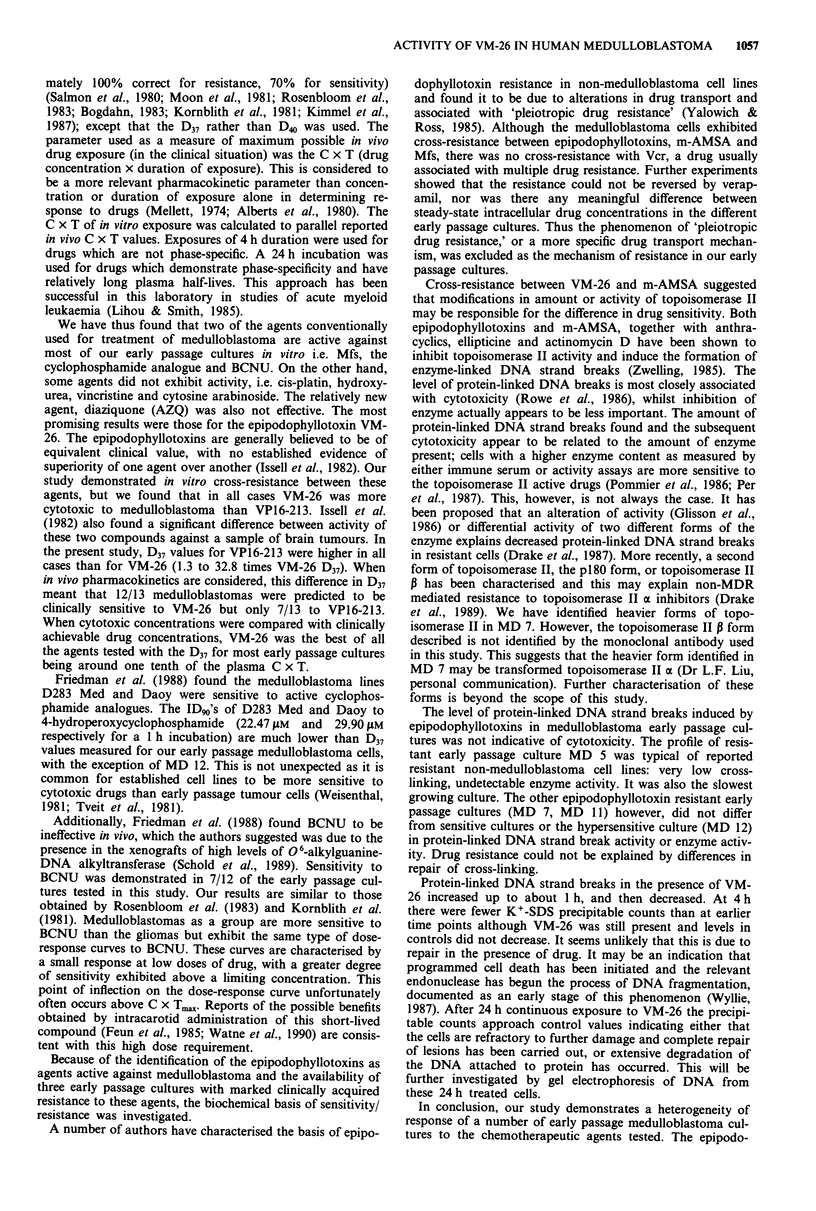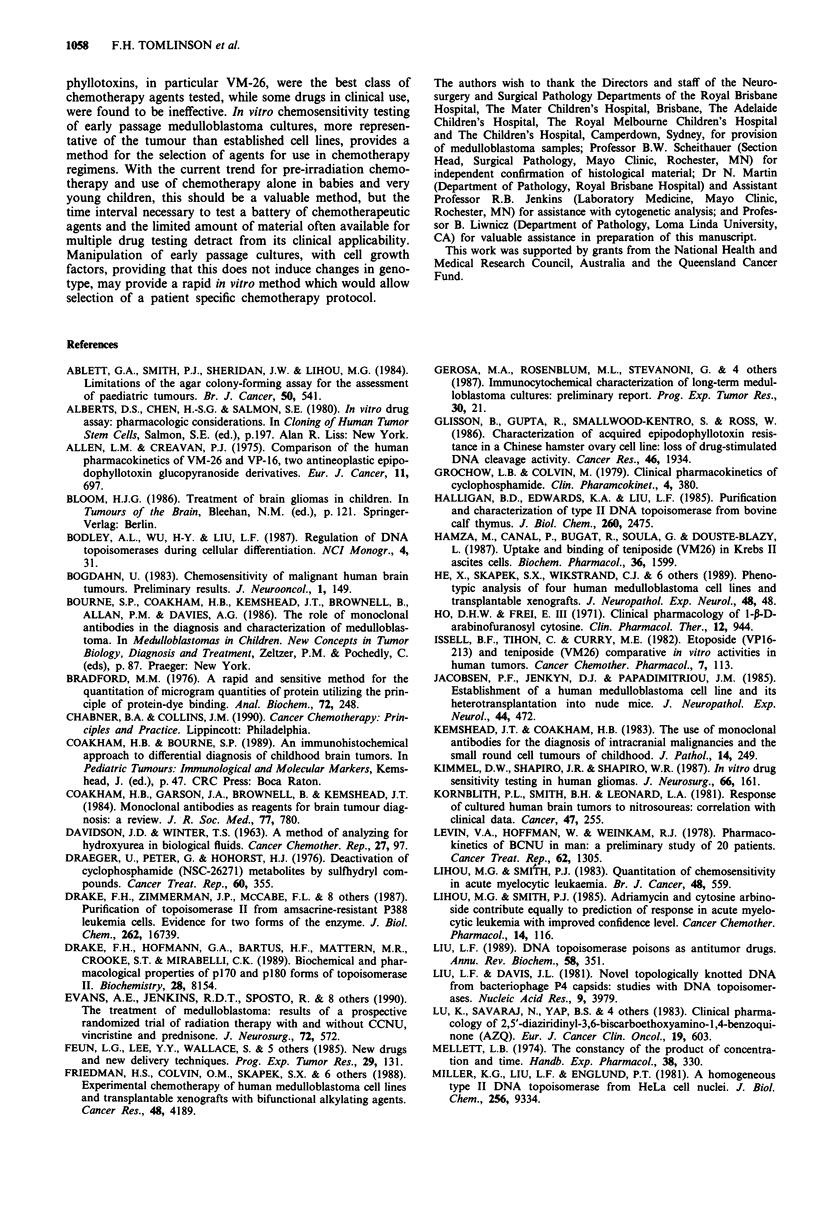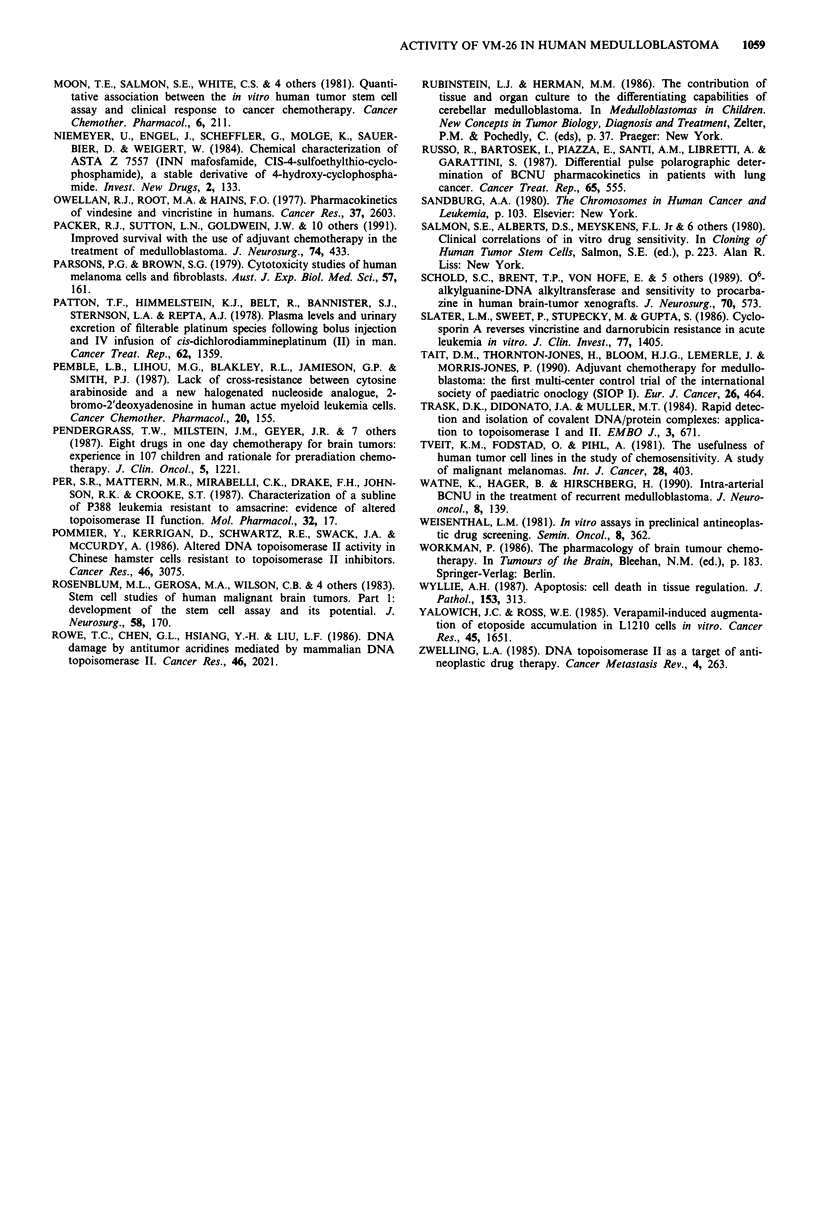# Comparison of in vitro activity of epipodophyllotoxins with other chemotherapeutic agents in human medulloblastomas.

**DOI:** 10.1038/bjc.1991.464

**Published:** 1991-12

**Authors:** F. H. Tomlinson, M. G. Lihou, P. J. Smith

**Affiliations:** Queensland Institute of Medical Research, Brisbane, Australia.

## Abstract

Surgical specimens from 15 medulloblastoma patients were used to establish early passage cultures. In vitro sensitivity to a battery of cytotoxic agents, including some in current medulloblastoma treatment protocols, was measured. Drug sensitivity was assessed at clinically relevant drug concentrations using the 3H-thymidine uptake method. Tumours were predicted to be sensitive if greater than 37% were killed by exposure to drugs at clinically achievable levels. A poor response to vincristine (Vcr), cis-platin (CDDP), hydroxyurea (HU) or diaziquone (AZQ) (no responders), and cytosine arabinoside (AraC) (1/12), was seen. Nine of ten tumours tested were sensitive to mafosfamide (Mfs); seven out of 12 were sensitive to carmustine (BCNU), 12 of 13 to teniposide (VM-26) and seven of 13 to etoposide (VP16-213). VM-26 was the best of the agents tested with most tumours responding to very low concentrations of drug, suggesting that the role of epipodophyllotoxins in treatment of brain tumours be further investigated. Despite the marked sensitivity of the medulloblastomas to the epipodophyllotoxins, three early passage cultures were much more resistant to these drugs than the average for the group. The basis of this resistance was investigated. Deficient cellular uptake of drug was excluded as a cause of resistance. One resistant early passage culture displayed low cellular activity of topoisomerase II and decreased levels of drug induced enzyme-DNA strand break activity. This was not the case for the other resistant early passage cultures: the basis of resistance in these cells does not appear to be due to any previously reported mechanism.


*Br. J. Cancer* (1991), **64**, 1051–1059 © Macmillan Press Ltd., 1991

### Comparison of *in vitro* activity of epipodophyllotoxins with other chemotherapeutic agents in human medulloblastomas

F.H. Tomlinson[1,2], M.G. Lihou[1] & P.J. Smith[1,3]

[1]*Queensland Institute of Medical Research, Brisbane, Australia 4006;* [2]*Department of Neurological Surgery, Mayo Clinic, Rochester, MN 55905, USA; and* [3]*Royal Children's Hospital, Brisbane, Australia 4006.*

**Summary** Surgical specimens from 15 medulloblastoma patients were used to establish early passage cultures. *In vitro* sensitivity to a battery of cytotoxic agents, including some in current medulloblastoma treatment protocols, was measured. Drug sensitivity was assessed at clinically relevant drug concentrations using the $^3$H-thymidine uptake method. Tumours were predicted to be sensitive if >37% were killed by exposure to drugs at clinically achievable levels. A poor response to vincristine (Vcr), cis-platin (CDDP), hydroxyurea (HU) or diaziquone (AZQ) (no responders), and cytosine arabinoside (AraC) (1/12), was seen.

Nine of ten tumours tested were sensitive to mafosfamide (Mfs); seven out of 12 were sensitive to carmustine (BCNU), 12 of 13 to teniposide (VM-26) and seven of 13 to etoposide (VP16-213). VM-26 was the best of the agents tested with most tumours responding to very low concentrations of drug, suggesting that the role of epipodophyllotoxins in treatment of brain tumours be further investigated.

Despite the marked sensitivity of the medulloblastomas to the epipodophyllotoxins, three early passage cultures were much more resistant to these drugs than the average for the group. The basis of this resistance was investigated. Deficient cellular uptake of drug was excluded as a cause of resistance. One resistant early passage culture displayed low cellular activity of topoisomerase II and decreased levels of drug induced enzyme-DNA strand break activity. This was not the case for the other resistant early passage cultures: the basis of resistance in these cells does not appear to be due to any previously reported mechanism.

Medulloblastoma is an important paediatric brain tumour because of its high incidence and malignant behaviour. The overall disease free survival at 5 years, in patients receiving surgery and craniospinal axis radiotherapy, is approximately 50%. This is considered to be the upper limit of curability by these treatment modalities. Chemotherapy has been shown to be advantageous in selected patient groups either as adjuvant therapy or as treatment of recurrent disease. The value of chemotherapy in the treatment of medulloblastoma will be dependent on the availability and identification of drugs active against this tumour. At present, protocols for chemotherapy usually include the lipophilic compounds carmustine (BCNU) or lomustine and procarbazine. Vincristine (Vcr), whilst poorly absorbed by cerebrospinal fluid and brain, is also commonly used (Bloom, 1986; Workman, 1986). Currently '8 in 1' therapy is being evaluated (Pendergrass *et al.*, 1987).

Cytotoxicity has been assessed by clonal assays in agar or on plastic, incorporation of $^3$H-thymidine into DNA, uptake of vital dyes and other methods with much the same results. Predictions of resistance are close to 100% correct; prediction of sensitivity, whilst less reliable, still has a success rate of around 70% (Salmon *et al.*, 1980; Moon *et al.*, 1981; Rosenbloom *et al.*, 1983; Bogdahn, 1983; Kornblith *et al.*, 1981; Kimmel *et al.*, 1987). We have examined drug sensitivity in human medulloblastoma by using 14 early passage cultures. Our study provides data on the degree of heterogeneity of response between medulloblastomas obtained from a number of patients. Furthermore, the cells were from early passage cultures, and hence should give a better indication of chemosensitivity of the tumours *in vivo*. The epipodophyllotoxins teniposide (VM-26) and etoposide (VP16-213) were identified as potentially useful agents in this disease. We investigated the basis of primary clinical resistance to these drugs by quantitating intracellular steady-state drug concentrations, drug-induced protein-linked DNA strand breaks, and topoisomerase II expression and activity.

Correspondence: F.H. Tomlinson, Department of Neurosurgery, Mayo Clinic, Rochester, Minnesota 55905, USA.
Received 2 January 1991; and in revised form 22 July 1991.

### Materials and methods

### *Establishment of cell cultures*

All studies were made on material collected at procedures performed for clinical reasons and remaining after sufficient tissue has been taken for clinical laboratory study. The histological diagnosis was confirmed by an independent neuropathologist (Professor B.W. Scheithauer). Over a period of 2 years, 16 medulloblastoma specimens were received from a total of 15 patients. The age of the patients ranged from 3 to 11 years (median 5.5 years) with a male to female ratio of 2:1. Most of the samples were from primary tumours however both a primary tumour (MD 6) and a metastasis removed at the same time (MD 6M) were obtained from one patient. A recurrent fourth ventricle tumour (MD 5) was removed 3 years after a patient had completed a full course of craniospinal irradiation and post irradiation chemotherapy and in another patient a frontal metastasis (MD 14) was removed 15 months following extirpation of the primary lesion and a full course of craniospinal irradiation.

Tumours samples were dissociated and cloned in agar as described by Ablett *et al.* (1984). Primary cultures, were established in tissue culture flasks from dissociated tumour cells in RPMI 1640 with 10% FCS, were incubated at 37°C in an environment of 5% $O_2$, 5% $CO_2$, 90% $N_2$ and 100% humidity. When the monolayers were almost confluent, the cultures were passed. Cultures were characterised around passage No. 4 and against around passage No. 10. Experiments were not performed on cells after 12 passages. They were routinely checked for mycoplasma.

### *Characterisation of early passage cultures*

The early passage cultures were characterised by immunohistochemical methods for neuron specific enolase (NSE) (Dako PAP kit, Dako Corp., Santa Barbara, USA) and by a panel of monoclonal antibodies (UJ13A and A2B5 which bind to normal and neoplastic neuroectodermal tissue, UJ181.4 which shows specificity to oncofoetal antigen expressed by foetal brain and neuroblastic tumours and UJ127.11 which recognises antigens of normal neuroectodermal tissue, neuronal tumours and schwannomas) all of which were kindly provided by Dr J.T. Kemshead (Brain Tumour Research Laboratory, Frenchay Hospital, Bristol, England)

(Coakham *et al.*, 1984). As frozen material was not available, immunohistochemical staining was not performed on the surgical specimen. Cytogenetic analysis was performed on early passage cultures as described by Sandburg (1980). Metaphases were G- banded (G- bands with Trypsin and Giemsa). At least five metaphases were examined per culture. DNA flow cytometry was performed as described by Pemble *et al.* (1987). Normal HeLa cells were used as control.

*Drug testing protocol*

A reduction in survival, at clinically achievable drug levels, to 37% was adopted for classifying tumours into sensitive and resistant groups (Salmon *et al.*, 1980; Moon *et al.*, 1981; Lihou & Smith, 1983 & 1985; Kornblith *et al.*, 1981; Rosenbloom *et al.*, 1983; Kimmel *et al.*, 1987). The pharmacokinetic parameter C × T (concentration time.product) (Mellett, 1974; Alberts *et al.*, 1980) was used as the measure of clinically achievable drug exposure. The C × T achieved in patients receiving standard drug doses was obtained from, or calculated from published data (Tables I, II). The *in vitro* C × T required to reduce survival to 37% was calculated simply by multiplying the $D_{37}$, the drug concentration required to reduce survival to 37% of control values, by the number of hours of incubation i.e. 24 h (Tables I, II). This was compared with the C × T achievable in patients (Tables I, II): tumours were classified as sensitive if the former figure was less than the latter.

The medulloblastoma cells were exposed for 4 h to drugs generally regarded as cycle specific, but not phase-specific (diaziquone (AZQ) cis-platin (CDDP), mafosfamide (Mfs), adriamycin (Adr)) (Chabner & Collins, 1990). Cells were exposed to BCNU (cycle-specific drug) for 24 h, since the drug has a short half-life ($t_{1/2}$). At 37°C, the $t_{1/2}$ of BCNU *in vitro* is 12 min (Russo *et al.*, 1987), hence the drug concentration is close to 0 after about 2 h. It was calculated that a 24 h incubation with 10 μM BCNU resulted in an *in vitro* C × T of 2.9 μM.h. The alkylating activity of Mfs *in vitro* was calculated assuming rapid hydrolysis to 4-OH cyclophosphamide (Niemeyer *et al.*, 1984) and an alkylating activity half-life for 4-OH cyclophosphamide *in vitro* of 3 h (Draeger *et al.*, 1976). A 4 h incubation with 20 μM Mfs results in a C × T for alkylating equivalents of 52 μM.h. Early passage cultures were exposed to the phase-specific agents i.e. cytosine arabinoside (AraC), hydroxyurea (HU), VM-26 and VP16-213 for 24 h, since the cells grew slowly. The doubling time up to the 10th passage was around 3 days for all early passage cultures except MD 12 (1.2 days).

**Table I** Sensitivity of medulloblastomas to phase specific agents

| | | | $D_{37}$ (μM)[a] | | |
|---|---|---|---|---|---|
| *Culture* | *Vcr* | *AraC* | *HU* | *VM-26* | *VP16-213* |
| MD 3 | 0.158 | 1.23 | 32,460 | 0.66 | 4.36 |
| MD 4 | 0.257 | 0.17 | 22,100 | 0.92 | 2.00 |
| MD 5 | 0.131 | 2.79 | 16,800 | 15.17 | 43.92 |
| MD 6 | 0.144 | 3.77 | 15,440 | 0.67 | 25.37 |
| MD 7 | 0.277 | – | 2,950 | 49.30 | 37.21 |
| MD 9 | 0.377 | 7.36 | 5,800 | 0.75 | 3.81 |
| MD 10 | 0.123 | 1.29 | – | 1.05 | 3.51 |
| MD 11 | 0.205 | 1.29 | 3,200 | 11.60 | 30.46 |
| MD 12 | 0.012 | 0.42 | 570 | 0.03 | 0.19 |
| MD 13 | 0.096 | 4.66 | 26,560 | 0.95 | 4.42 |
| MD 14 | 0.060 | 2.40 | 3,010 | 0.20 | 1.44 |
| MD 15 | 0.053 | 2.58 | 50,330 | 0.20 | 0.41 |
| MD 17 | 0.469 | 0.83 | 2,960 | 0.16 | 0.58 |
| Sensitive[b] | 0/13 | 1/12 | 0/11 | 12/13 | 7/13 |

Dose response curves were repeated 3–9 times for each culture and each agent. The mean value of response at each dose was calculated together with the standard error (s.e.m.) which ranged from 5–15% of the mean value. [a]$D_{37}$ values were calculated from these curves by linear regression analysis. [b]Tumour cell lines were classified as sensitive to an agent if $D_{37}$ × duration of exposure was less than the clinically achievable C × T in patients. *In vivo* pharmacokinetic parameters of dose (mg $m^{-2}$), C × T (μM.h) and peak plasma concentration (μM) are as follows: vincristine (Vcr) – 1.5, 0.11 and 0.70 (Owellan *et al.*, 1977); cytosine arabinoside (AraC) – 300, 17.7, 22.6 (Ho & Frei, 1971); hydroxyurea (HU) – 3000 p.o, 1703, 191 (Davidson & Winter, 1963); teniposide (VM-26) – 165, 370, 51.3 (Allen & Creavan, 1975) and etoposide (VP16-213) – 100, 103, 17.7 (Allen & Creaven, 1975). – Not done.

**Table II** Sensitivity of medulloblastomas to BCNU, Mfs, CDDP, AZQ

| | | $D_{37}$ (μM)[a] | | |
|---|---|---|---|---|
| *Culture* | *BCNU* | *Mfs* | *CDDP* | *AZQ* |
| MD 3 | 7.64 | 42.49 | 7.71 | 288 |
| MD 4 | 8.80 | 14.56 | 8.80 | 294 |
| MD 5 | – | 52.92 | 4.54 | 420 |
| MD 6 | 31.41 | 51.66 | 5.30 | 66 |
| MD 7 | 30.25 | 83.41 | 9.14 | 350 |
| MD 9 | 2.64 | 29.40 | 3.20 | – |
| MD 10 | 8.68 | 15.85 | 7.88 | 63 |
| MD 11 | 23.48 | 47.74 | 7.44 | 166 |
| MD 12 | 7.24 | 3.60 | 1.80 | – |
| MD 13 | 9.75 | – | 6.16 | 26 |
| MD 14 | 9.36 | – | – | – |
| MD 15 | 12.07 | – | 5.31 | – |
| MD 17 | 33.00 | – | 16.93 | – |
| Sensitive[b] | 7/12 | 9/10 | 0/12 | 0/8 |

[a]$D_{37}$ was calculated as outlined for Table I. The $D_{37}$ values given above were calculated from the initial drug concentration in the incubation. These figures have not been adjusted to take into account activity decay. [b]Tumours were classified as sensitive or resistant as described for Table I. The $D_{37}$ × duration of exposure values were calculated assuming a $t_{1/2}$ for BCNU of 12 min and rapid hydrolysis of mafosfamide (Mfs) to 4-OH-cyclophosphamide and $t_{1/2}$ for alkylating activity for this compound of 3 h. *In vivo* pharmacokinetic parameters of dose (mg $m^{-2}$), C × T (μM.h) and peak plasma concentration (μM) are as follows: carmustine (BCNU) – 60, 3.1, 6.0 (Levin *et al.*, 1978); Mfs, derived data from cyclophosphamide pharmacokinetic dose and C × T values – 1,200, 1,280 (Grochow & Colvin, 1979). Expressed as alkylating equivalents C × T and peak plasma concentration values are 139 and 27; cis-platin (CDDP) – 90, 5.9, 7.5 (Patton *et al.*, 1978) and diaziquone (AZQ) – 9, 4.07 and 0.37 (Lu *et al.*, 1983). – Not done.

*Cell survival*

Cell survival was determined by $^3$H-thymidine incorporation. Early passage cultures were seeded in 0.1 ml of medium, in 96-well microtitre plates 24 h before treatment with drug (six replicates for each drug concentration) at a cell concentration of $4 \times 10^4$ $ml^{-1}$. The cells were exposed to drugs whilst attached to the bottom of wells in microtitre plates. Control untreated cells were examined daily until almost confluent. Five to 7 days after treatment was initiated, the medium was replaced with medium containing [$^3$H-methyl]-thymidine (5 μCi $ml^{-1}$, 40 Ci $mmol^{-1}$; RadioChemical Centre, Amersham, UK). After 24 h at 37°C, the cells were washed with Dulbecco's solution, detached with 1.25 mg $ml^{-1}$ pancreatin (Sigma, St Louis, USA), 0.16 M NaCl, 6 mM $Na_2HPO_4$, 2.7 mM KCl, 1.5 mM $KH_2PO_4$ and 6.0 mM EDTA, pH 7.2, filtered onto glass fibre discs (Whatman GF/C), and lysed and washed exhaustively with water prior to liquid scintillation counting. Survivals were compared on the basis of $D_{37}$, using Spearman Rank correlation analysis.

*Intracellular VP16-213 concentration*

Intracellular accumulation of [$^3$H]VP16-213 was studied using the modified method of Hamza *et al.* (1987) with fewer cells per replicate ($10^5$ cells/ml/replicate) and substituting VP16-213 (10 μM) (900 mCi $mmol^{-1}$; Moravek Biochemicals, Brea, USA) for VM-26, as radiolabelled VM-26 was unavailable. Initially, cellular uptake was measured at time points ranging from 5 to 120 min at an extracellular [$^3$H]VP16-213 concentration of 10 μM. The time-course of uptake was followed in three medulloblastoma early passage cultures (MD 11, 12 and 14). Cellular uptake of [$^3$H]VP16-213 at 120 min was used to compare differences between nine medulloblastoma early passage cultures.

*Detection of protein-linked DNA strand breaks*

The SDS-$K^+$ precipitation method used was a modification of that developed by Trask *et al.* (1984) and used to detect drug damage by Rowe *et al.* (1986). All early passage cultures used were harvested in log-phase growth. Cells were labelled overnight with 0.8 μM $ml^{-1}$ of $^3$H-thymidine (specific activity 41 Ci $mmol^{-1}$) and cells ($2 \times 10^4$ cells/vial) rested for 1 h at 37°C in a gassed (5% $O_2$, 5% $CO_2$, 90% $N_2$), humidified incubator. Drug was then added and cells incubated for the required period. At different time points, the suspension was diluted with 5 volumes Dulbecco's medium and spun at $150 \times g$ for 10 min. Liquid scintillation counting was performed following transfer of the pellet resuspended in 200 μl of water at 65°C into scintillation tubes containing 3.5 ml of Instagel (Packard, Ill., USA). Additionally, cross-link repair was determined by further incubation in drug free media.

*Preparation of nuclear lysate*

Nuclear lysates were prepared according to the method of Miller *et al.* (1981) but as limited numbers of cells were available the method was scaled down 100-fold. Between $10^6$ and $10^7$ cells were used to make the lysates which were stored at −20°C following dialysis against 30 mM potassium phosphate pH 7.0, 50% glycerol, 0.5 mM dithiothreitol and 0.1 mM EDTA.

*Topoisomerase II activity*

Topoisomerase II activity of nuclear lysate from medulloblastoma early passage cultures was assessed using the P4 DNA unknotting assay (Liu & Davis, 1981). Incubation was performed for 1 h. The protein content of nuclear lysates was measured by the Coomassie blue method of Bradford (1976).

*Topoisomerase II expression*

The topoisomerase II content was assessed by immunoblotting of cell extracts using anti-topoisomerase II antisera (Halligan *et al.*, 1985). The topoisomerase II antibody was kindly donated by Dr L.F. Liu (Johns Hopkins University, School of Medicine, Baltimore, USA). Murine erythroleukaemia cells (MELC), clone 745 of C. Friend, also donated by Dr L.F. Liu, were used as controls.

### Results

In vitro *growth*

All tumours plated in agar formed colonies (40 cells or more) after 2 to 4 weeks culture. The cloning efficiency in agar, of the four primary cultures tested, was very low (MD 4, 0.010 ± 0.001; MD 5, 0.0011 ± 0.0005; MD 7, 0.0023 ± 0.0009; MD 9, 0.0004 ± 0.0003; MD 10, 0.0015 ± 0.0002; mean ± standard deviation). Most of the specimens provided were not of sufficient size for plating in agar and therefore primary cultures from these tumours were established in liquid culture on plastic. Fourteen of 16 samples, including a primary tumour and spinal metastasis, were established in liquid culture.

*Characterisation of early passage cultures*

All early passage cultures were positive for the following antibodies: UJ127.11 which recognises antigens on normal ectodermal tissue, neuronal tumours and schwannomas; UJ13A and A2B5 which recognise normal and neoplastic neuroectodermal tissue; and UJ181.4 which recognises an oncofoetal antigen expressed by foetal brain and neuroblastic tumours. Staining for NSE was negative in all early passage cultures. Karyotyping was successfully performed in eight out of ten early passage cultures of which four were abnormal (Table III). Of the four specimens submitted for flow cytometry, two were aneuploid (Table III).

**Table III** Cytogenetic and DNA flow-cytometric analysis of medulloblastoma early passage cultures

| *Cell culture* | *Karyotype* | *DNA ploidy*[a] |
|---|---|---|
| MD 3 | No metaphases | NA |
| MD 4 | 48,XY, + 8, + 21,del(4)(p14) | NA |
| MD 5 | 46,XY, − 10, − 10,12p + ,t(3;14)(q21;q24), + 2mar | NA |
| MD 6 | No metaphases | NA |
| MD 7 | NA | Aneuploid |
| MD 9 | 46,XX | NA |
| MD 10 | 46,Y, − X,der(X)t(X;1) (p23.3;q21) | NA |
| MD 11 | NA | Diploid |
| MD 12 | 48,XY, + Y, − 13,der(1)t(1;?)(p36.3;?), der(11)t(11;?)(q23.1;?), del(17)(p11.2), der(6)t(6;8)(p11.2;q22), + mar | Diploid |
| MD 13 | 46,XY | NA |
| MD 14 | NA | Aneuploid |
| MD 15 | 46,XY | NA |
| MD 17 | 46,XY | NA |

[a]Early passage cells with a DNA histogram indistinguishable from normal HeLa cells were classified as diploid, however, in the case of MD 12, karyotypic analysis indicated additional chromosomes were present. NA not available.

*Proliferative activity of early passage cultures*

Although the experiments were performed at the earliest possible passages, the variation in control $^3$H-Thymidine incorporation between experiments was still quite high, decreasing as the cultures became older. Despite this variation, a reliable estimate of drug sensitivity was obtained. The standard error of the mean of survival at a given drug concentration obtained for three to ten experiments was usually less than 10%. The average control values for incorporated d.p.m. are given in Figure 1. This average was taken from all cytotoxicity experiments (approximately 30 to 100 independent experiments per culture, 12 control wells per experiment).

*Sensitivity to phase-specific agents*

All three phase-specific agents in the '8 in 1' protocol (Pendergrass *et al.*, 1987) were tested i.c. AraC, HU and Vcr, and the calculated $D_{37}$ values are given in Table I. There was a general trend for faster growing cells to be more sensitive to the phase-specific agents. However, Spearman rank correlation analysis was performed for each set of data and no significant association was found between drug sensitivity and d.p.m. in control cultures of the same experiments, indicating that factors other than proliferative status also influenced sensitivity to these agents.

There was a heterogeneous response to the phase-specific agents, with the highest $D_{37}$ being 40 times (Vcr), 43 times

**Figure 1** DPM incorporated into control cultures. The average d.p.m. incorporated into each control well are given above. This average was taken from all cytotoxicity experiments reported in this paper, this being approximately 30 to 100 independent experiments per culture. In each experiment at least six control wells were used.

(AraC) or 88 times (HU) higher than the lowest measured. The fastest growing early passage culture, MD 12, was by far the most sensitive to all three agents, but no culture was consistently more resistant than others. Spearman rank correlation analysis revealed no significant cross-sensitivity between any combination of these agents. This again indicates that factors other than those affecting cell growth determined *in vitro* sensitivity.

Only one tumour was considered sensitive to clinically achievable levels of AraC and none to either HU or Vcr (Table I). A very high concentration of HU was required to kill cells when compared with plasma drug levels.

*Sensitivity to epipodophyllotoxins*

Similar experiments to those described above were carried out using the epipodophyllotoxins VM-26 and VP16-213, nonintercalative topoisomerase II poisons (Liu, 1989). The response was heterogenous (Table I); the range of response to VM-26 being greater than that for any other drug tested. The *in vitro* response was not related to d.p.m. in control cultures, but sensitivity to VM-26 was highly, significantly correlated with response to VP16-213 ($r = 0.852$, $P < 0.002$, $n = 13$) (Table IV).

Twelve of 13 (92%) tumours were considered sensitive to clinically achievable levels of VM-26 as were seven out of 13 (54%) to VP16-213 (Table I). This difference in responsiveness was due to two factors; on a molar basis, VM-26 was far more cytotoxic than VP16-213, and the plasma pharmacokinetics reported for VP16-213 are worse than for VM-26, because lower doses of VP16-213 must be administered and for the same dose the C × T and the peak plasma concentration are lower (Table I). Most medulloblastoma cells were very sensitive to VM-26. Ten of the sensitive early passage cultures responded at levels which were less than 1/10 of that achievable in patients and for one culture (MD 12) this figure was 1/600. In this regard, VM-26 was the best agent tested in this study.

Three early passage cultures however, were markedly more resistant to VM-26 than average. The $D_{37}$ values for these cultures were 15.17 µM for MD5; 49.30 µM; for MD 7 and 11.60 µM for MD 11. These cells were approximately 400–1600 times as resistant as the most sensitive culture (MD 12, $D_{37}$ 0.03 µM) and 20 to 80 times more resistant than average for the remaining cultures ($D_{37}$ 0.62 µM ± 0.35 µM s.d). The same three early passage cultures were also resistant to VP16-213. The difference between sensitive and resistant cultures was not as marked as for VM-26, for $D_{37}$ for the VP16-213 resistant cultures being around 300 times that of MD 12 and 13 times the average for the other nine early passage cultures.

*Sensitivity to alkylating and cross-linking agents*

Medulloblastoma early passage cultures were also tested for sensitivity to alkylating and cross-linking agents i.e. BCNU, Mfs, CDDP and AZQ (Table II). A spectrum of responses was observed but the differences between the most sensitive and most resistant cultures ($D_{37}$ resistant cultures/$D_{37}$ sensitive culture) were less for this group of agents than for the phase-specific agents. These ratios were five for BCNU, 23 for Mfs, 9 for CDDP and 16 for AZQ.

Early passage cultures resistant to the epipodophyllotoxins (MD 5, MD 6, MD 7 and MD 11) were generally more resistant than average to alkylating agents as well. MD 6 however appeared to be relatively sensitive to AZQ. Again, MD 12 was the most sensitive to all these agents, but as mentioned above, the differences between cultures were not as great for this class of agents.

None of the tumours were sensitive to CDDP or AZQ but seven of 12 were sensitive to BCNU and nine out of ten were sensitive to Mfs (Table II). In all cases, the level of *in vitro* drug exposure had to be of the same order of magnitude as the maximum clinically achievable level to reduce survival to 37% of control, untreated cells. In other words, none of the tumours tested were very sensitive to any agent from this group.

*Sensitivity to amsacrine (m-AMSA)*

Sensitivity to a DNA intercalating topoisomerase II poison (M-AMSA) (Liu, 1989) was tested (Figure 2). The range of responses to this agent was large, the highest $D_{37}$ calculated being 399 times the lowest. MD 5 and MD7 were very resistant to this agent, whilst MD 11 was only marginally more resistant than average. Rank correlation analysis revealed significant relationships between sensitivity to this agent and epidodophyllotoxins, and also with Mfs (Table IV).

*Patterns of cross-resistance and collateral sensitivity*

As stated previously, the data in Tables I and II was analysed for cross-resistance and collateral sensitivity relationships by the Spearman rank correlation test (Table IV). Cross resistance between VM-26 and VP16-213 was confirmed. There was also a significant relationship between resistance to these agents and to the other topoisomerase II poison tested, m-AMSA.

There was no apparent relationship between sensitivity to either VM-26, VP16-213 or m-AMSA and Vcr suggesting that the phenomenon of 'pleiotropic drug resistance' may not be responsible for the patterns of cross-resistance seen. Dose-response experiments with VM-26 were also performed in the presence of agents known to reverse this type of resistance: verapamil (10 µM) (Yalowich & Ross, 1985) and cyclosporin A (7 and 13 $\mu g\ ml^{-1}$) (Slater *et al.*, 1986). Neither of these agents significantly altered sensitivity to VM-26 in any of the early passage cultures tested; all cultures were tested except for MD 10 and MD 17. A surprising finding was the association between Mfs sensitivity and response to either VP16-213 or m-AMSA. The relationship with VM-26 was not significant. The only agent for which sensitivity was significantly linked with the amount of $^3$H-thymidine incorporation in control cells was AZQ. There was no significant difference between results obtained from MD 6, the primary tumour and MD 6M, a metastasis obtained from the same patient.

**Table IV** Spearman rank correlation analysis of *in vitro* response data

| *Variables* | *r*[a] | *P* | *No.*[b] |
|---|---|---|---|
| VM-26 & VP16-213 | 0.852 | <0.002 | 13 |
| VP16-213 & ASTA | 0.902 | <0.002 | 10 |
| m-AMSA & VM-26 | 0.888 | <0.002 | 12 |
| m-AMSA & VP16-213 | 0.839 | <0.002 | 12 |
| m-AMSA & ASTA | 0.685 | <0.05 | 10 |
| AZQ & control d.p.m. | 0.982 | <0.05 | 8 |

The data given in Tables I and II were analysed for cross-sensitivity relationships between different agents. All possible combinations were tested, and in addition the relationship between response to a drug and the d.p.m. counted in control, untreated plates (ie. an indication of the proliferative status of the cells) was also tested. [a]r, the probability of there being a positive relationship between sensitivity to two different agents, or between an agent and the proliferative status of control cells. [b]Number of early passage cultures tested.

**Figure 2** Sensitivity of medulloblastoma to m-AMSA. [a]$D_{37}$values were calculated as outlined for Table I.

### *Intracellular accumulation of VP16-213*

Cellular uptake in medulloblastoma early passage cultures measured at time points ranging from 5 to 120 min demonstrated that steady-state was achieved in all cultures by 60 min (Figure 3). Cellular uptake at 120 min in nine medulloblastoma cultures ranged from 4.88 to 20.26 pmol $10^{-5}$ cells (Table V). The most resistant cultures fell in the middle of this range (MD 5, 9.04; MD 7, 15.69; MD 11, 10.30 pmoles $10^{-5}$ cells), whilst the hypersensitive culture, MD 12, had one of the lowest values. Estimation of cell diameters allowed calculation of the cellular volume and thus the intracellular drug concentration. Cell diameters ranged from 17.8 to 19.9 μm. The standard deviation ranged from 5% to 8% of the mean, indicating no significant difference between cell diameters of different early passage cultures. It was therefore calculated that all of these cultures were capable of concentrating the drug. When the steady-state drug concentration was compared with cellular drug sensitivity, no correlation was found.

**Figure 4** Protein-linked DNA strand break activity following the 1 h incubation with 1 μM to 100 μM VM-26. The mean ± s.e.m. were calculated for each time point for 3–8 experiments. Within each experiment duplicate or triplicate determinations were performed.

### *Protein-linked DNA strand break activity*

Results were expressed as the ratio of the d.p.m. precipitated in the presence of drug over d.p.m. precipitated without drug (control). The fact that some precipitable counts were measured in the absence of drug is an indication of the basal level of topoisomerase II activity present. A range of VM-26 concentrations from 1 μM to 100 μM were used initially (Figure 4) and tested in six early passage cultures. Protein-linked DNA strand breaks were found to occur at all concentrations tested. Maximum protein-linked DNA strand break activity occurred at 30 μM VM-26 except in one of the cultures tested (MD 6), where maximum breaks occurred at 70 μM. Above these concentrations, the number of protein-linked DNA strand breaks decreased in spite of the higher drug concentration.

**Figure 3** Intracellular accumulation of VP16-213. Cellular uptake of three medulloblastoma (MD) early passage cultures measured at time points ranging from 5 to 120 min at an extracellular [$^3$H]VP16-213 concentration of 10 μM. Three experiments testing each in duplicate were performed each culture. The mean value ± s.e.m. was calculated for each time point.

**Table V** Cellular uptake of [$^3$H]VP16-213 in medullobastoma cells

| *Culture* | *Uptake (moles $10^5$ cells × $10^{-12}$)* | *Cell diameter (μM)* | *Cell volume (1 × $10^{-12}$)* | *Intracellular $^3$H VP16-213 (μM)* |
|---|---|---|---|---|
| MD 4 | 5.82 ± 0.35 | 18.90 | 3.54 | 16.44 ± 0.99 |
| MD 5 | 9.04 ± 2.10 | 18.60 | 3.37 | 26.82 ± 6.23 |
| MD 7 | 15.69 ± 0.29 | 19.90 | 4.13 | 37.99 ± 0.70 |
| MD 11 | 10.30 ± 2.71 | 17.80 | 2.95 | 34.92 ± 9.19 |
| MD 12 | 6.24 ± 1.16 | 17.90 | 3.00 | 20.80 ± 3.87 |
| MD 13 | 20.26 ± 1.53 | 18.50 | 3.32 | 61.02 ± 4.61 |
| MD 14 | 5.60 ± 0.76 | 18.20 | 3.16 | 17.72 ± 2.40 |
| MD 15 | 4.88 ± 0.41 | 18.10 | 3.10 | 15.74 ± 1.32 |
| MD 17 | 14.46 ± 2.31 | 18.40 | 3.26 | 44.36 ± 7.09 |

Uptake was measured after a 2 h incubation with [$^3$H]VP16-213 at 37°C. Duplicate determinations were performed for each experiment. Mean uptake was calculated from the results from three experiments expressed as ± SEM.

Protein-linked DNA strand breaks were compared in all available medulloblastoma early passage cultures following a 1 h incubation with 10 μM and 30 μM VM-26. The latter concentration was chosen since this was likely to result in maximum DNA damage due to protein-linked DNA strand breaks, is a clinically achievable concentration and is highly cytotoxic in the $^3$H-thymidine assay. At the lower concentration of 10 μM VM-26, the number of protein-linked DNA strand breaks was elevated by a factor of approximately ten times control. At 30 μM VM-26, the number of protein-linked DNA strand breaks was elevated by a factor of around 20 times control. Some cultures exhibited less protein-linked DNA strand break activity. MD 5, in particular, was resistant to this form of drug-induced damage. No overall correlation between epipodophyllotoxin sensitivity and induction of protein-linked DNA strand breaks was apparent.

In medulloblastoma early passage cultures, VM-26 was much more cytotoxic to cells on an equimolar basis than VP16-213 (Table I). Experiments were performed to compare the number of protein-linked DNA strand breaks produced by VP16-213 and VM-26 in four medulloblastoma cultures. Two of the four medulloblastoma early passage culture (MD 7, MD 11) were resistant to epipodophyllotoxins. The number of protein-linked DNA strand breaks produced by VM-26 was approximately 1.8 times greater than that produced by VP16-213.

### *Protein–linked DNA strand break time-course*

Production of protein-linked DNA strand breaks by continuous exposure to VM-26 (10 μM and 30 μM), was measured in medulloblastoma early passage cultures for time points 10 min to 24 h (Figure 5). Generally, maximum protein-linked DNA strand break frequency occurred at 1 h for both drug concentrations. The time-course of induction of protein-linked DNA strand breaks at 30 μM VM-26 in the VM-26 resistant cultures MD 7 and MD 11 was no different from that of the sensitive lines MD 12, MD 14. In the VM-26 resistant culture MD 5, however, the frequency of protein-linked DNA strand breaks did not vary with exposure duration. In spite of the continuous presence of VM-26, at time points after 1 h, d.p.m. precipitated by SDS-$K^+$ approached control values.

After long term exposure to VM-26 (24 h) the frequency of protein-linked DNA strand breaks at 10 μM and 30 μM concentrations was not significantly different from control values for early passage cultures other than MD 7. In this culture, the frequency of protein-linked DNA strand breaks was 5.1 and 8.4 times control at 10 μM and 30 μM respectively.

**Figure 5** Protein-linked DNA strand break activity produced over 24 h following incubation with **a**, 10 μM and **b**, 30 μM VM-26. The mean ± s.e.m. were calculated for each time point for 3–8 experiments. Within each experiment duplicate or triplicate determinations were performed.

*mAMSA-induced protein-linked DNA strand breaks*

Protein-linked DNA strand break activity was examined following exposure to m-AMSA (3 μg ml$^{-1}$ for 1 h and 24 h). This concentration was chosen because it was a clinically achievable level and resulted in significant cytotoxicity (being of the order of the $D_{37}$ for the medulloblastoma group). At this concentration m-AMSA was readily soluble in RPMI 1640 10% FCS. At 1 h, significant protein-linked DNA strand break activity was observed which was roughly equivalent to that produced by 10 μM VM-26. Again, few links were observed in the MD 5 early passage culture. After 24 h, protein-linked DNA strand breaks were still apparent but were lower than at 1 h.

*Time course of protein-linked DNA strand break repair*

Repair of protein-linked DNA strand breaks was investigated in medulloblastoma early passage cultures (MD 7, 11, 12, 14, 15 and 17) after incubation with 30 μM VM-26 for 1 h. After washing, SDS-KCl precipitable d.p.m. were measured at time-points ranging from 15 min to 24 h. The time-course of repair of protein-linked DNA strand breaks was rapid and similar for all cultures. Values approached control within 1 h after removal of drug. In the VM-26 resistant culture MD 7, values fell below control at 3 h. At 24 h values for all cultures approximated control.

*Topoisomerase II activity*

Topoisomerase II activity in nuclear lysates from MD 5, 7, 12 and 14 cells was assayed using a P4 unknotting assay. Extracts from MD 12 had the greatest amount of activity, detectable with as little as 0.1 μg nuclear protein. Activity in the other early passage cultures was much lower than MD 12 with detectable activity at 1 μg protein for MD 11 and 3 μg for both MD 7 and MD 14. There was no detectable activity in nuclear lysates from MD 5 cells.

*Topoisomerase II expression*

In three medulloblastoma early passage cultures, MD 7, MD 12, MD 14, a band of similar molecular weight to topoisomerase II (170 kDa) previously characterised in mouse friend leukaemia cells (Bodley *et al.*, 1987; personal communication L.F. Liu, 1990), was identified. Consistent with the results of the activity assay, the amount of enzyme was high in MD 12 cells and much lower in the other two cultures, with more immunoreactive protein in MD 7 than MD 14. Insufficient cells were available to enable enzyme expression in MD 5 and MD 11. Two bands of high molecular weight greater than 180 kDa were recognised in the MD 7 cells but not the other lines. A number of bands of lower molecular weight were seen in all early passage cultures and presumably represent fragmented protein.

### Discussion

The benefits of surgery and radiotherapy for treatment of malignant disease are generally considered to be approaching the maximal attainable limits. Further improvement in disease free survival may be achieved by chemotherapy but there is considerable heterogeneity of response to different agents; both between patients and between tumour types. The present study concentrated on medulloblastoma; a tumour with a poor prognosis but where chemotherapy has been shown to be advantageous for some patients (Evans *et al.*, 1990; Tait *et al.*, 1990; Packer *et al.*, 1991; Bloom, 1986).

We have examined the *in vitro* sensitivity of early passaged cultures from 13 medulloblastoma patients to a number of drugs. Since the cultures have not been extensively passage, the possibility of selection from an originally heterogenous sample of tumour cells is therefore diminished. The results should give an indication of the degree of heterogeneity of response between different tumours.

The clinical criteria for selection of tumour samples for use in this study were based on: (a) demographic characteristics and clinical presentation; (b) the radiologic appearance; (c) the histologic appearance. The identity of the cultured cells was determined on morphological (Rubinstein & Herman, 1986; Gerosa *et al.*, 1987) immunohistochemical, cytogenetic and DNA flow cytometric analysis. The immunohistochemical profile was determined using a panel of murine monoclonal antibodies (UJ13A, UJ181.4, UJ127.11 and A2B5). These antibodies have been successfully used for the characterisation of surgical material and established cell lines (Kemshead & Coakham, 1983; Bourne *et al.*, 1986; He *et al.*, 1989; Coakham & Bourne, 1989).

As with surgical specimens of gliomas (Rosenbloom *et al.*, 1983), medulloblastoma primary cultures exhibited poor cloning efficiencies in semisolid media in contrast to the high cloning efficiency seen in an established medulloblastoma cell line Daoy (Jacobsen *et al.*, 1985). However, medulloblastoma is unlike gliomas in that clonal growth on plastic is hard to assess due to cell mobility and poor growth at low cell density. In this study, we used an end-point of cellular $^3$H-thymidine uptake 5–7 days after drug exposure as an indicator of drug toxicity. This has been found to correlate with clonal growth in agar (Parsons & Brown, 1979).

The cut-off used for sensitivity in the present study was that found by others to predict clinical response (approximately 100% correct for resistance, 70% for sensitivity) (Salmon *et al.*, 1980; Moon *et al.*, 1981; Rosenbloom *et al.*, 1983; Bogdahn, 1983; Kornblith *et al.*, 1981; Kimmel *et al.*, 1987); except that the $D_{37}$ rather than $D_{40}$ was used. The parameter used as a measure of maximum possible *in vivo* drug exposure (in the clinical situation) was the C × T (drug concentration × duration of exposure). This is considered to be a more relevant pharmacokinetic parameter than concentration or duration of exposure alone in determining response to drugs (Mellett, 1974; Alberts *et al.*, 1980). The C × T of *in vitro* exposure was calculated to parallel reported *in vivo* C × T values. Exposures of 4 h duration were used for drugs which are not phase-specific. A 24 h incubation was used for drugs which demonstrate phase-specificity and have relatively long plasma half-lives. This approach has been successful in this laboratory in studies of acute myeloid leukaemia (Lihou & Smith, 1985).

We have thus found that two of the agents conventionally used for treatment of medulloblastoma are active against most of our early passage cultures *in vitro* i.e. Mfs, the cyclophosphamide analogue and BCNU. On the other hand, some agents did not exhibit activity, i.e. cis-platin, hydroxyurea, vincristine and cytosine arabinoside. The relatively new agent, diaziquone (AZQ) was also not effective. The most promising results were those for the epipodophyllotoxin VM-26. The epipodophyllotoxins are generally believed to be of equivalent clinical value, with no established evidence of superiority of one agent over another (Issell *et al.*, 1982). Our study demonstrated *in vitro* cross-resistance between these agents, but we found that in all cases VM-26 was more cytotoxic to medulloblastoma than VP16-213. Issell *et al.* (1982) also found a significant difference between activity of these two compounds against a sample of brain tumours. In the present study, $D_{37}$ values for VP16-213 were higher in all cases than for VM-26 (1.3 to 32.8 times VM-26 $D_{37}$). When *in vivo* pharmacokinetics are considered, this difference in $D_{37}$ meant that 12/13 medulloblastomas were predicted to be clinically sensitive to VM-26 but only 7/13 to VP16-213. When cytotoxic concentrations were compared with clinically achievable drug concentrations, VM-26 was the best of all the agents tested with the $D_{37}$ for most early passage cultures being around one tenth of the plasma C × T.

Friedman *et al.* (1988) found the medulloblastoma lines D283 Med and Daoy were sensitive to active cyclophosphamide analogues. The $ID_{90}$'s of D283 Med and Daoy to 4-hydroperoxycyclophosphamide (22.47 μM and 29.90 μM respectively for a 1 h incubation) are much lower than $D_{37}$ values measured for our early passage medulloblastoma cells, with the exception of MD 12. This is not unexpected as it is common for established cell lines to be more sensitive to cytotoxic drugs than early passage tumour cells (Weisenthal, 1981; Tveit *et al.*, 1981).

Additionally, Friedman *et al.* (1988) found BCNU to be ineffective *in vivo*, which the authors suggested was due to the presence in the xenografts of high levels of $O^6$-alkylguanine-DNA alkyltransferase (Schold *et al.*, 1989). Sensitivity to BCNU was demonstrated in 7/12 of the early passage cultures tested in this study. Our results are similar to those obtained by Rosenbloom *et al.* (1983) and Kornblith *et al.* (1981). Medulloblastomas as a group are more sensitive to BCNU than the gliomas but exhibit the same type of dose-response curves to BCNU. These curves are characterised by a small response at low doses of drug, with a greater degree of sensitivity exhibited above a limiting concentration. This point of inflection on the dose-response curve unfortunately often occurs above C × $T_{max}$. Reports of the possible benefits obtained by intracarotid administration of this short-lived compound (Feun *et al.*, 1985; Watne *et al.*, 1990) are consistent with this high dose requirement.

Because of the identification of the epipodophyllotoxins as agents active against medulloblastoma and the availability of three early passage cultures with marked clinically acquired resistance to these agents, the biochemical basis of sensitivity/resistance was investigated.

A number of authors have characterised the basis of epipodophyllotoxin resistance in non-medulloblastoma cell lines and found it to be due to alterations in drug transport and associated with 'pleiotropic drug resistance' (Yalowich & Ross, 1985). Although the medulloblastoma cells exhibited cross-resistance between epipodophyllotoxins, m-AMSA and Mfs, there was no cross-resistance with Vcr, a drug usually associated with multiple drug resistance. Further experiments showed that the resistance could not be reversed by verapamil, nor was there any meaningful difference between steady-state intracellular drug concentrations in the different early passage cultures. Thus the phenomenon of 'pleiotropic drug resistance,' or a more specific drug transport mechanism, was excluded as the mechanism of resistance in our early passage cultures.

Cross-resistance between VM-26 and m-AMSA suggested that modifications in amount or activity of topoisomerase II may be responsible for the difference in drug sensitivity. Both epipodophyllotoxins and m-AMSA, together with anthracyclics, ellipticine and actinomycin D have been shown to inhibit topoisomerase II activity and induce the formation of enzyme-linked DNA strand breaks (Zwelling, 1985). The level of protein-linked DNA breaks is most closely associated with cytotoxicity (Rowe *et al.*, 1986), whilst inhibition of enzyme actually appears to be less important. The amount of protein-linked DNA strand breaks found and the subsequent cytotoxicity appear to be related to the amount of enzyme present; cells with a higher enzyme content as measured by either immune serum or activity assays are more sensitive to the topoisomerase II active drugs (Pommier *et al.*, 1986; Per *et al.*, 1987). This, however, is not always the case. It has been proposed that an alteration of activity (Glisson *et al.*, 1986) or differential activity of two different forms of the enzyme explains decreased protein-linked DNA strand breaks in resistant cells (Drake *et al.*, 1987). More recently, a second form of topoisomerase II, the p180 form, or topoisomerase II β has been characterised and this may explain non-MDR mediated resistance to topoisomerase II α inhibitors (Drake *et al.*, 1989). We have identified heavier forms of topoisomerase II in MD 7. However, the topoisomerase II β form described is not identified by the monoclonal antibody used in this study. This suggests that the heavier form identified in MD 7 may be transformed topoisomerase II α (Dr L.F. Liu, personal communication). Further characterisation of these forms is beyond the scope of this study.

The level of protein-linked DNA strand breaks induced by epipodophyllotoxins in medulloblastoma early passage cultures was not indicative of cytotoxicity. The profile of resistant early passage culture MD 5 was typical of reported resistant non-medulloblastoma cell lines: very low cross-linking, undetectable enzyme activity. It was also the slowest growing culture. The other epipodophyllotoxin resistant early passage cultures (MD 7, MD 11) however, did not differ from sensitive cultures or the hypersensitive culture (MD 12) in protein-linked DNA strand break activity or enzyme activity. Drug resistance could not be explained by differences in repair of cross-linking.

Protein-linked DNA strand breaks in the presence of VM-26 increased up to about 1 h, and then decreased. At 4 h there were fewer $K^+$-SDS precipitable counts than at earlier time points although VM-26 was still present and levels in controls did not decrease. It seems unlikely that this is due to repair in the presence of drug. It may be an indication that programmed cell death has been initiated and the relevant endonuclease has begun the process of DNA fragmentation, documented as an early stage of this phenomenon (Wyllie, 1987). After 24 h continuous exposure to VM-26 the precipitable counts approach control values indicating either that the cells are refractory to further damage and complete repair of lesions has been carried out, or extensive degradation of the DNA attached to protein has occurred. This will be further investigated by gel electrophoresis of DNA from these 24 h treated cells.

In conclusion, our study demonstrates a heterogeneity of response of a number of early passage medulloblastoma cultures to the chemotherapeutic agents tested. The epipodophyllotoxins, in particular VM-26, were the best class of chemotherapy agents tested, while some drugs in clinical use, were found to be ineffective. *In vitro* chemosensitivity testing of early passage medulloblastoma cultures, more representative of the tumour than established cell lines, provides a method for the selection of agents for use in chemotherapy regimens. With the current trend for pre-irradiation chemotherapy and use of chemotherapy alone in babies and very young children, this should be a valuable method, but the time interval necessary to test a battery of chemotherapeutic agents and the limited amount of material often available for multiple drug testing detract from its clinical applicability. Manipulation of early passage cultures, with cell growth factors, providing that this does not induce changes in genotype, may provide a rapid *in vitro* method which would allow selection of a patient specific chemotherapy protocol.

The authors wish to thank the Directors and staff of the Neurosurgery and Surgical Pathology Departments of the Royal Brisbane Hospital, The Mater Children's Hospital, Brisbane, The Adelaide Children's Hospital, The Royal Melbourne Children's Hospital and The Children's Hospital, Camperdown, Sydney, for provision of medulloblastoma samples; Professor B.W. Scheithauer (Section Head, Surgical Pathology, Mayo Clinic, Rochester, MN) for independent confirmation of histological material; Dr N. Martin (Department of Pathology, Royal Brisbane Hospital) and Assistant Professor R.B. Jenkins (Laboratory Medicine, Mayo Clinic, Rochester, MN) for assistance with cytogenetic analysis; and Professor B. Liwnicz (Department of Pathology, Loma Linda University, CA) for valuable assistance in preparation of this manuscript.

This work was supported by grants from the National Health and Medical Research Council, Australia and the Queensland Cancer Fund.

### References

ABLETT, G.A., SMITH, P.J., SHERIDAN, J.W. & LIHOU, M.G. (1984). Limitations of the agar colony-forming assay for the assessment of paediatric tumours. *Br. J. Cancer*, **50**, 541.

ALBERTS, D.S., CHEN, H.-S.G. & SALMON, S.E. (1980). *In vitro* drug assay: pharmacologic considerations. In *Cloning of Human Tumor Stem Cells*, Salmon, S.E. (ed.), p.197. Alan R. Liss: New York.

ALLEN, L.M. & CREAVAN, P.J. (1975). Comparison of the human pharmacokinetics of VM-26 and VP-16, two antineoplastic epipodophyllotoxin glucopyranoside derivatives. *Eur. J. Cancer*, **11**, 697.

BLOOM, H.J.G. (1986). Treatment of brain gliomas in children. In *Tumours of the Brain*, Bleehan, N.M. (ed.), p. 121. Springer-Verlag: Berlin.

BODLEY, A.L., WU, H-Y. & LIU, L.F. (1987). Regulation of DNA topoisomerases during cellular differentiation. *NCI Monogr.*, **4**, 31.

BOGDAHN, U. (1983). Chemosensitivity of malignant human brain tumours. Preliminary results. *J. Neurooncol.*, **1**, 149.

BOURNE, S.P., COAKHAM, H.B., KEMSHEAD, J.T., BROWNELL, B., ALLAN, P.M. & DAVIES, A.G. (1986). The role of monoclonal antibodies in the diagnosis and characterization of medulloblastoma. In *Medulloblastomas in Children. New Concepts in Tumor Biology, Diagnosis and Treatment*, Zeltzer, P.M. & Pochedly, C. (eds), p. 87. Praeger: New York.

BRADFORD, M.M. (1976). A rapid and sensitive method for the quantitation of microgram quantities of protein utilizing the principle of protein-dye binding. *Anal. Biochem.*, **72**, 248.

CHABNER, B.A. & COLLINS, J.M. (1990). *Cancer Chemotherapy: Principles and Practice*. Lippincott: Philadelphia.

COAKHAM, H.B. & BOURNE, S.P. (1989). An immunohistochemical approach to differential diagnosis of childhood brain tumors. In *Pediatric Tumours: Immunological and Molecular Markers*, Kemshead, J. (ed.), p. 47. CRC Press: Boca Raton.

COAKHAM, H.B., GARSON, J.A., BROWNELL, B. & KEMSHEAD, J.T. (1984). Monoclonal antibodies as reagents for brain tumour diagnosis: a review. *J. R. Soc. Med.*, **77**, 780.

DAVIDSON, J.D. & WINTER, T.S. (1963). A method of analyzing for hydroxyurea in biological fluids. *Cancer Chemother. Rep.*, **27**, 97.

DRAEGER, U., PETER, G. & HOHORST, H.J. (1976). Deactivation of cyclophosphamide (NSC-26271) metabolites by sulfhydryl compounds. *Cancer Treat. Rep.*, **60**, 355.

DRAKE, F.H., ZIMMERMAN, J.P., McCABE, F.L. & 8 others (1987). Purification of topoisomerase II from amsacrine-resistant P388 leukemia cells. Evidence for two forms of the enzyme. *J. Biol. Chem.*, **262**, 16739.

DRAKE, F.H., HOFMANN, G.A., BARTUS, H.F., MATTERN, M.R., CROOKE, S.T. & MIRABELLI, C.K. (1989). Biochemical and pharmacological properties of p170 and p180 forms of topoisomerase II. *Biochemistry*, **28**, 8154.

EVANS, A.E., JENKINS, R.D.T., SPOSTO, R. & 8 others (1990). The treatment of medulloblastoma: results of a prospective randomized trial of radiation therapy with and without CCNU, vincristine and prednisone. *J. Neurosurg.*, **72**, 572.

FEUN, L.G., LEE, Y.Y., WALLACE, S. & 5 others (1985). New drugs and new delivery techniques. *Prog. Exp. Tumor Res.*, **29**, 131.

FRIEDMAN, H.S., COLVIN, O.M., SKAPEK, S.X. & 6 others (1988). Experimental chemotherapy of human medulloblastoma cell lines and transplantable xenografts with bifunctional alkylating agents. *Cancer Res.*, **48**, 4189.

GEROSA, M.A., ROSENBLUM, M.L., STEVANONI, G. & 4 others (1987). Immunocytochemical characterization of long-term medulloblastoma cultures: preliminary report. *Prog. Exp. Tumor Res.*, **30**, 21.

GLISSON, B., GUPTA, R., SMALLWOOD-KENTRO, S. & ROSS, W. (1986). Characterization of acquired epipodophyllotoxin resistance in a Chinese hamster ovary cell line: loss of drug-stimulated DNA cleavage activity. *Cancer Res.*, **46**, 1934.

GROCHOW, L.B. & COLVIN, M. (1979). Clinical pharmacokinetics of cyclophosphamide. *Clin. Pharamcokinet.*, **4**, 380.

HALLIGAN, B.D., EDWARDS, K.A. & LIU, L.F. (1985). Purification and characterization of type II DNA topoisomerase from bovine calf thymus. *J. Biol. Chem.*, **260**, 2475.

HAMZA, M., CANAL, P., BUGAT, R., SOULA, G. & DOUSTE-BLAZY, L. (1987). Uptake and binding of teniposide (VM26) in Krebs II ascites cells. *Biochem. Pharmacol.*, **36**, 1599.

HE, X., SKAPEK, S.X., WIKSTRAND, C.J. & 6 others (1989). Phenotypic analysis of four human medulloblastoma cell lines and transplantable xenografts. *J. Neuropathol. Exp. Neurol.*, **48**, 48.

HO, D.H.W. & FREI, E. III (1971). Clinical pharmacology of 1-$\beta$-D-arabinofuranosyl cytosine. *Clin. Pharmacol. Ther.*, **12**, 944.

ISSELL, B.F., TIHON, C. & CURRY, M.E. (1982). Etoposide (VP16-213) and teniposide (VM26) comparative *in vitro* activities in human tumors. *Cancer Chemother. Pharmacol.*, **7**, 113.

JACOBSEN, P.F., JENKYN, D.J. & PAPADIMITRIOU, J.M. (1985). Establishment of a human medulloblastoma cell line and its heterotransplantation into nude mice. *J. Neuropathol. Exp. Neurol.*, **44**, 472.

KEMSHEAD, J.T. & COAKHAM, H.B. (1983). The use of monoclonal antibodies for the diagnosis of intracranial malignancies and the small round cell tumours of childhood. *J. Pathol.*, **14**, 249.

KIMMEL, D.W., SHAPIRO, J.R. & SHAPIRO, W.R. (1987). *In vitro* drug sensitivity testing in human gliomas. *J. Neurosurg.*, **66**, 161.

KORNBLITH, P.L., SMITH, B.H. & LEONARD, L.A. (1981). Response of cultured human brain tumors to nitrosoureas: correlation with clinical data. *Cancer*, **47**, 255.

LEVIN, V.A., HOFFMAN, W. & WEINKAM, R.J. (1978). Pharmacokinetics of BCNU in man: a preliminary study of 20 patients. *Cancer Treat. Rep.*, **62**, 1305.

LIHOU, M.G. & SMITH, P.J. (1983). Quantitation of chemosensitivity in acute myelocytic leukaemia. *Br. J. Cancer*, **48**, 559.

LIHOU, M.G. & SMITH, P.J. (1985). Adriamycin and cytosine arbinoside contribute equally to prediction of response in acute myelocytic leukemia with improved confidence level. *Cancer Chemother. Pharmacol.*, **14**, 116.

LIU, L.F. (1989). DNA topoisomerase poisons as antitumor drugs. *Annu. Rev. Biochem.*, **58**, 351.

LIU, L.F. & DAVIS, J.L. (1981). Novel topologically knotted DNA from bacteriophage P4 capsids: studies with DNA topoisomerases. *Nucleic Acid Res.*, **9**, 3979.

LU, K., SAVARAJ, N., YAP, B.S. & 4 others (1983). Clinical pharmacology of 2,5′-diaziridinyl-3,6-biscarboethoxyamino-1,4-benzoquinone (AZQ). *Eur. J. Cancer Clin. Oncol.*, **19**, 603.

MELLETT, L.B. (1974). The constancy of the product of concentration and time. *Handb. Exp. Pharmacol.*, **38**, 330.

MILLER, K.G., LIU, L.F. & ENGLUND, P.T. (1981). A homogeneous type II DNA topoisomerase from HeLa cell nuclei. *J. Biol. Chem.*, **256**, 9334.

MOON, T.E., SALMON, S.E., WHITE, C.S. & 4 others (1981). Quantitative association between the *in vitro* human tumor stem cell assay and clinical response to cancer chemotherapy. *Cancer Chemother. Pharmacol.*, **6**, 211.

NIEMEYER, U., ENGEL, J., SCHEFFLER, G., MOLGE, K., SAUERBIER, D. & WEIGERT, W. (1984). Chemical characterization of ASTA Z 7557 (INN mafosfamide, CIS-4-sulfoethylthio-cyclophosphamide), a stable derivative of 4-hydroxy-cyclophosphamide. *Invest. New Drugs*, **2**, 133.

OWELLAN, R.J., ROOT, M.A. & HAINS, F.O. (1977). Pharmacokinetics of vindesine and vincristine in humans. *Cancer Res.*, **37**, 2603.

PACKER, R.J., SUTTON, L.N., GOLDWEIN, J.W. & 10 others (1991). Improved survival with the use of adjuvant chemotherapy in the treatment of medulloblastoma. *J. Neurosurg.*, **74**, 433.

PARSONS, P.G. & BROWN, S.G. (1979). Cytotoxicity studies of human melanoma cells and fibroblasts. *Aust. J. Exp. Biol. Med. Sci.*, **57**, 161.

PATTON, T.F., HIMMELSTEIN, K.J., BELT, R., BANNISTER, S.J., STERNSON, L.A. & REPTA, A.J. (1978). Plasma levels and urinary excretion of filterable platinum species following bolus injection and IV infusion of *cis*-dichlorodiammineplatinum (II) in man. *Cancer Treat. Rep.*, **62**, 1359.

PEMBLE, L.B., LIHOU, M.G., BLAKLEY, R.L., JAMIESON, G.P. & SMITH, P.J. (1987). Lack of cross-resistance between cytosine arabinoside and a new halogenated nucleoside analogue, 2-bromo-2'deoxyadenosine in human actue myeloid leukemia cells. *Cancer Chemother. Pharmacol.*, **20**, 155.

PENDERGRASS, T.W., MILSTEIN, J.M., GEYER, J.R. & 7 others (1987). Eight drugs in one day chemotherapy for brain tumors: experience in 107 children and rationale for preradiation chemotherapy. *J. Clin. Oncol.*, **5**, 1221.

PER, S.R., MATTERN, M.R., MIRABELLI, C.K., DRAKE, F.H., JOHNSON, R.K. & CROOKE, S.T. (1987). Characterization of a subline of P388 leukemia resistant to amsacrine: evidence of altered topoisomerase II function. *Mol. Pharmacol.*, **32**, 17.

POMMIER, Y., KERRIGAN, D., SCHWARTZ, R.E., SWACK, J.A. & MCCURDY, A. (1986). Altered DNA topoisomerase II activity in Chinese hamster cells resistant to topoisomerase II inhibitors. *Cancer Res.*, **46**, 3075.

ROSENBLUM, M.L., GEROSA, M.A., WILSON, C.B. & 4 others (1983). Stem cell studies of human malignant brain tumors. Part 1: development of the stem cell assay and its potential. *J. Neurosurg.*, **58**, 170.

ROWE, T.C., CHEN, G.L., HSIANG, Y.-H. & LIU, L.F. (1986). DNA damage by antitumor acridines mediated by mammalian DNA topoisomerase II. *Cancer Res.*, **46**, 2021.

RUBINSTEIN, L.J. & HERMAN, M.M. (1986). The contribution of tissue and organ culture to the differentiating capabilities of cerebellar medulloblastoma. In *Medulloblastomas in Children. New Concepts in Tumor Biology, Diagnosis and Treatment*, Zelter, P.M. & Pochedly, C. (eds), p. 37. Praeger: New York.

RUSSO, R., BARTOSEK, I., PIAZZA, E., SANTI, A.M., LIBRETTI, A. & GARATTINI, S. (1987). Differential pulse polarographic determination of BCNU pharmacokinetics in patients with lung cancer. *Cancer Treat. Rep.*, **65**, 555.

SANDBURG, A.A. (1980). *The Chromosomes in Human Cancer and Leukemia*, p. 103. Elsevier: New York.

SALMON, S.E., ALBERTS, D.S., MEYSKENS, F.L. Jr & 6 others (1980). Clinical correlations of in vitro drug sensitivity. In *Cloning of Human Tumor Stem Cells*, Salmon, S.E. (ed.), p. 223. Alan R. Liss: New York.

SCHOLD, S.C., BRENT, T.P., VON HOFE, E. & 5 others (1989). $O^6$-alkylguanine-DNA alkyltransferase and sensitivity to procarbazine in human brain-tumor xenografts. *J. Neurosurg.*, **70**, 573.

SLATER, L.M., SWEET, P., STUPECKY, M. & GUPTA, S. (1986). Cyclosporin A reverses vincristine and darnorubicin resistance in acute leukemia *in vitro*. *J. Clin. Invest.*, **77**, 1405.

TAIT, D.M., THORNTON-JONES, H., BLOOM, H.J.G., LEMERLE, J. & MORRIS-JONES, P. (1990). Adjuvant chemotherapy for medulloblastoma: the first multi-center control trial of the international society of paediatric onoclogy (SIOP I). *Eur. J. Cancer*, **26**, 464.

TRASK, D.K., DIDONATO, J.A. & MULLER, M.T. (1984). Rapid detection and isolation of covalent DNA/protein complexes: application to topoisomerase I and II. *EMBO J.*, **3**, 671.

TVEIT, K.M., FODSTAD, O. & PIHL, A. (1981). The usefulness of human tumor cell lines in the study of chemosensitivity. A study of malignant melanomas. *Int. J. Cancer*, **28**, 403.

WATNE, K., HAGER, B. & HIRSCHBERG, H. (1990). Intra-arterial BCNU in the treatment of recurrent medulloblastoma. *J. Neuro-oncol.*, **8**, 139.

WEISENTHAL, L.M. (1981). *In vitro* assays in preclinical antineoplastic drug screening. *Semin. Oncol.*, **8**, 362.

WORKMAN, P. (1986). The pharmacology of brain tumour chemotherapy. In *Tumours of the Brain*, Bleehan, N.M. (ed.), p. 183. Springer-Verlag: Berlin.

WYLLIE, A.H. (1987). Apoptosis: cell death in tissue regulation. *J. Pathol.*, **153**, 313.

YALOWICH, J.C. & ROSS, W.E. (1985). Verapamil-induced augmentation of etoposide accumulation in L1210 cells *in vitro*. *Cancer Res.*, **45**, 1651.

ZWELLING, L.A. (1985). DNA topoisomerase II as a target of antineoplastic drug therapy. *Cancer Metastasis Rev.*, **4**, 263.